# The role of craving in gambling behavior: examining its relevance and links to psychological distress, personality, and demographics

**DOI:** 10.1186/s40359-025-03816-4

**Published:** 2025-12-12

**Authors:** Thomas Westerberg, Anders Håkansson, Kristina Berglund

**Affiliations:** 1 Department of Psychology, University of Gothenburg, Gothenburg, Sweden; 2 Unit for Clinical Addiction Research, University of Lund, Malmo, Sweden

**Keywords:** Craving, Gambling problems, Affects, Mental health symptoms, Personality traits, Gender, Age

## Abstract

**Background:**

Several studies have sought to investigate gambling craving and its potential contribution to gambling problems. One aim of this line of research has been to further examine whether craving should be considered a separate diagnostic criterion for gambling disorder, which it currently is not. The present study aimed to investigate the relationship between craving and other risk factors—such as mental health symptoms, affect, and personality traits—and to determine whether craving makes a unique contribution to problematic gambling when controlling for these factors.

**Methods:**

In total, 1053 Swedish adult gamblers (55% male, median age 48.4 years) from a citizen panel completed a web survey including: Gambling Craving Scale (subscales *desire*, *relief*, and *anticipation*), Diagnostic Screen for Gambling Problems (NODS), and other scales. According to NODS, classification, 81% were non-problem gamblers, 11% at-risk gamblers, 4% problem gamblers, and 4% were classified as having probable gambling disorder. We conducted Spearman correlation analysis of the relationship of craving with gambling severity and other risk factors, and logistic regression exploring craving’s impact in problematic gamblers while controlling for risk factors.

**Results:**

The three craving subscales were strongly associated with gambling severity and weakly–moderately associated with mental health symptoms, negative affects, and the personality traits impulsivity, negative affectivity, antagonism, and alexithymia; the subscales were slightly–moderately negatively associated with positive affects and the personality trait hedonic capacity. When exploring craving’s impact on problematic gambling, controlling for mental health symptoms, affects, personality traits, age, and gender, the subscales *desire* and *anticipation*, alongside impulsivity and being male, increased the odds of problematic gambling.

**Conclusion:**

These findings suggests that craving, alongside impulsivity and male gender, is central to understanding problematic gambling, implying that prevention and treatment efforts should address these targets, and further support the inclusion of craving in future revision of diagnostic criteria for gambling disorder.

**Supplementary Information:**

The online version contains supplementary material available at 10.1186/s40359-025-03816-4.

## Introduction

The gambling landscape has undergone substantial changes in recent years with an unprecedented surge in gambling opportunities, given the advent of internet and mobile gambling [13, 69, 102]. While most individuals who engage in gambling do so non-problematically, some individuals become excessively preoccupied with gambling and cannot control their behavior despite severe negative social, financial, and health-related consequences [2, 52]. One hypothesis is that difficulty in the self-regulation of craving plays a pivotal role in the inability to inhibit harmful gambling behaviors and in the development and maintenance of gambling disorder [4, 73, 105]. However, despite being extensively studied in recent years, there is still no agreement on the definition of craving and its significance remains poorly understood.

Craving, or the synonymously used concepts urge, desire, or drive, is a dynamic state that varies over time [6, 51]. It can be described as an intense and compelling impulse to engage in one’s addictive behavior despite knowledge of its negative consequences [15, 59]. According to some scholars, craving plays a predictive role in relation to severity of the gambling problem [10, 21, 51]. These assumptions are also consistent with both theoretical perspectives [27, 94] and empirical findings [37, 68, 73], similar to what has been demonstrated in relation to substance use disorders (e.g., [50, 104]). This motivational driving force is derived from internal states (e.g., negative affects, escape from negative emotions, positive affects, euphoria, joy, and excitement) as well as external environmental circumstances (e.g., gambling-related cues, situations, and places). Despite uncertainty in gambling outcome (i.e., win or loss), the state can be triggered by motivational cues comprising anticipation of relief from negative affects as well as anticipation of positive affects [16, 27, 94]. Based on current findings, we conceptualize craving as a construct with various dimensions referring not only to desire as a means of experiencing positive affects but also to the desire to escape negative affects, together with an anticipatory component, for which an expectation of experiencing pleasure is created [24, 125].

Craving is a well-known risk factor for the inability to inhibit harmful gambling behaviors and in the development and maintenance of a gambling disorder; in addition, research shows that personality traits, affects, and mental health symptoms are significantly associated with disordered gambling. Regarding personality traits, people with gambling problems tend to exhibit higher levels of neuroticism, agreeableness, and openness, lower conscientiousness, and either higher or lower extraversion scores [31, 38, 65, 108]. Moreover, several studies have highlighted significant challenges concerning affects, particularly difficulties in emotional regulation, along with increased levels of anxiety and depressive symptoms in individuals with gambling disorders (e.g., [9, 78, 81]). In the light of these findings, personality traits, affects (e.g., emotional regulation difficulties), and higher levels of anxiety as well as depression symptoms might be important predictors of gambling problems.

Several studies have investigated specific aspects of gambling craving and its potential contribution to gambling problems in greater detail. One aim of this line of research has been to further discuss whether craving should be considered a separate diagnostic criterion for gambling disorder, which it currently is not (see the fifth edition of *Diagnostic and Statistical Manual of Mental Disorders*, [34]). Furthermore, studies have examined the relative contribution of craving to gambling problems while controlling for other factors associated with gambling problems, as described above (e.g., [21, 27, 33, 51]). This has been done to explore the relationship between craving and variables such as personality traits, affects, and psychological symptoms, as well as to assess its unique contribution to the severity of gambling problems. However, to our knowledge, only one study [112] has simultaneously examined the relationship among craving, personality traits, affects, and symptoms of mental health problems. In that study, the authors compared pathological gamblers (PGs) with alcohol-dependent subjects (ADSs), finding that PGs scored higher on craving measures and novelty seeking than did ADSs. Additionally, in PGs, craving was correlated positively with depression and negatively with both abstinence duration and reward dependence. The authors concluded that PGs experienced more intense craving than did ADSs. In the study by Tavares et al. [112], gambling craving was examined as a unidimensional construct, however, more recent research has suggested that this is not the case [16, 27, 73, 74]. A more recent study [25] examined the connections among various dimensions of craving, gambling severity, personality traits, and alcohol use in adolescents. Compared with adolescents who did not gamble and those who gambled without any problems, those with at-risk and problem gambling exhibited higher scores on craving subscales and for maladaptive personality traits and alcohol use. More specifically, the study showed that craving subscales for anticipation and desire, alongside antagonism, disinhibition, and alcohol use, were the most accurate predictors of adolescent gambling involvement. While these studies provide valuable insights, limitations remain. Ciccarelli et al. [25] focused solely on adolescents and did not address anxiety and depression symptoms or affects, while Tavares et al. [112] overlooked craving as a multidimensional construct.

Studies have obtained diverse results concerning how individual variables (e.g., personality traits, affective states, psychopathology, and psychological vulnerabilities) are associated with craving (e.g., [25, 73, 74]). However, these studies have usually focused on these variables in isolation from one another, this fragmented approach has limited our understanding of their potential interplay. Consequently, there is a notable gap in the literature regarding studies that concurrently explore how craving, personality traits, anxiety symptoms, depression symptoms, and affects, jointly relate to gambling problems.

The objective of this study was therefore to examine: 1) craving and its relationship with gambling severity, mental health symptoms, affects, and personality traits and 2) whether craving is associated with problematic gambling (at-risk gambling, problem gambling, gambling disorder) when controlling for mental health symptoms, affects, and personality traits, age, and gender.

## Materials and methods

### Participants

The data were collected from a web survey distributed to a citizen panel of the Swedish market research company Ipsos (https://www.ipsos.com/sv-se). Participants in the citizen panel had previously enrolled with their personal information in the Ipsos system, agreeing to receive surveys by e-mail. The company recruited individuals until representative distribution was obtained. Participants in this study (aged 18 years and older) had responded to a screening question, indicating that they had gambled for money within the past 12 months. The current data collection was conducted between May and August 2023, with 15–20 min being required to complete the survey. Before the data were obtained from Ipsos, all cases were weighted by gender and age to enhance representativeness and ensure more reliable analyses and results. The participants comprised 1053 Swedish adult gamblers (54.9% male) aged 18–84 years (*M* = 48.42, *SD* = 17.29).

### Procedure

This study involved participants who had voluntarily enrolled in a citizen panel maintained for research purposes. Individuals from this panel were invited to participate in the present study. Eligibility for inclusion required that participants had engaged in gambling activities within the past twelve months. Participation was entirely voluntary, and informed consent was obtained from all respondents prior to data collection. The study has been conducted in accordance with the Swedish Act concerning the Ethical Review of Research Involving Humans [101]:460) and approved by the Swedish Ethical Review Authority (approval no. 2022–06330-01) on 13 January 2023. This Act, which is consistent with the principles of the WMA Declaration of Helsinki [123], underscores the protection of individuals rights, interests, and well-being throughout the research process. All participants received comprehensive written information about the purpose, procedures, and potential risks and benefits of the study. Participants were informed that they retained the right to withdraw from the study at any point without the need to provide a justification, and that such withdrawal would carry no adverse consequences. All data were handled confidentially and anonymized to ensure the protection of participants’ privacy. In acknowledgement of their participation in this study, individuals earned credits in the company’s bonus system.

### Measures

The web survey consisted of six sections, as described below.

*Sociodemographic and gambling-related* variables were age, gender identity, marital status, education level, domestic or foreign origin (defined as born or not born in Sweden, having one or both parents not born in Sweden), income-generating occupation, monthly income before taxes, gambling modality (e.g., Internet/online or not), type of gambling (e.g., sports betting, poker games, and lotteries), and gambling frequency (e.g., not gambled, last year, last month, and last week or more).

The Swedish version of the *NORC Diagnostic Screen for Gambling Problems – Past Year* version (NODS-SA; [43], translated into Swedish by [83]) was used for assessing gambling problem severity during the previous 12 months. NODS-SA is a 17-item self-report measure designed to assess gambling disorder as diagnostically defined in DSM-IV, using a dichotomous yes–no format [53]. Total score, which ranges from 0 to 10, is derived by summing “yes” responses to the designated items: [1 or 2], 3, 5, 7, [8 or 9], 10, 12, 13, [14 or 15 or 16], and 17. Each item within a cluster (1/2, 8/9, and 14/15/16) is counted as a single point, while items 4, 6, and 11 are excluded from scoring. Higher scores indicate greater gambling severity, with a score of 1 or 2 indicating at-risk gambling, of 3 or 4 problem gambling, and of 5 or more pathological gambling. Gerstein et al. [43] reported excellent test–retest reliability for the NODS (*r* = 0.99, *p* = 0.01) and good convergent validity. NODS-SA as used here was found to have excellent internal consistency (Cronbach’s α = 0.92, 95% CI [0.91–0.92]).

Gambling craving was measured with the *Gambling Craving Scale* (GACS; [125]), consisting of nine items, with three sub-scales. Each sub-scale contains three items: (1) an intention to gamble that was anticipated to be fun and enjoyable (Anticipation); (2) strong, urgent desire to gamble (Desire); and (3) relief from negative experiences expected from gambling (Relief). Each item is rated on a seven-point Likert scale ranging from 1 (strong disagreement) to 7 (strong agreement). For each scale, the three item responses are summed and divided by three to calculate a mean scale score. Higher scores indicate higher levels of craving for gambling. In previous studies, GACS has shown good psychometric properties (⍺ = 0.65–0.87 for anticipation, 0.81–0.85 for relief, and 0.81–0.89 for desire) in measuring different dimensions of gambling craving [15, 37, 125]. In the present sample, the internal consistency values for anticipation of 0.655 (95% CI [0.617–0.690]), for relief of 0.779 (95% CI [0.775–0.801]), and for desire of 0.851 (95% CI [0.851–0.866]) ranged from adequate to very good.

To evaluate anxiety and depression symptoms, the *Hospital Anxiety and Depression Scale* (HADS; [126]) was used. HADS is a 14-item self-report instrument consisting of seven items assessing anxiety (HADS-A) and seven items assessing depression (HADS-D). All items are rated on an ordinal response scale having four response categories (0–3), with higher scores representing greater symptom severity. Studies using HADS have recurrently been found reliable and valid for independently assessing depression and anxiety symptoms (HADS-A ⍺ = 0.80–0.93 and HADS-D ⍺ = 0.81–0.90) in clinical samples of patients and in broader populations [106, 126]. In the present study sample, Cronbach’s α was very good, close to excellent for both the anxiety scale (0.877, 95% CI [0.865–0.888]) and depression scale (0.843, 95% CI [0.828–0.857]).

Positive and negative affects were assessed using the *Positive and Negative Affect Scales* (PANAS; [117]). The ten-item PANAS scales were created to provide simple, reliable, and valid measures of the two higher-order dimensions of self-rated affect. The Positive Affect (PA) scale consists of the terms active, alert, attentive, determined, enthusiastic, excited, inspired, interested, proud, and strong, whereas the terms comprising the Negative Affect (NA) scale are afraid, ashamed, distressed, guilty, hostile, irritable, jittery, nervous, scared, and upset*.* Respondents rate the extent to which they have experienced each term on a five-point scale on which 1 = *very slightly or not at all*, 2 = *a little*, 3 = *moderately*, 4 = *quite a bit*, and 5 = *very much.* High levels of reliability and validity have been reported for these scales used in assessments, with Cronbach’s α values for NA of 0.84–0.87 and for PA of 0.86–0.90 [30, 117, 118]. In the present study, the internal consistency of each scale was excellent: PA had a Cronbach’s α of 0.896 (95% CI [0.886–0.905]) and NA had a Cronbach’s α of 0.922 (95% CI [0.915–0.929]).

Personality traits were assessed using the *Health-Relevant Personality Inventory* (Hp5i; [49]), a self-rating instrument based on selected facets corresponding to those of the five-factor model: agreeableness, contentiousness, neuroticism, extraversion, and openness [48]. Hp5i consists of 20 items grouped into five facet scales (i.e., four items per scale) and rated on four-point Likert scales ranging from 1 (*does not apply at all*) to 4 (*applies completely*). The five scales are labeled: antagonism (a facet of agreeableness), impulsivity (a facet of conscientiousness), hedonic capacity (a facet of extraversion), negative affectivity (a facet of neuroticism), and alexithymia (a facet of openness). Three independent studies assessing Hp5i subscales on different samples [47–49] have estimated fair to very good reliability values of 0.65–0.74 for antagonism, 0.66–0.81 for impulsivity, 0.54–0.80 for hedonic capacity, 0.67–0.70 for negative affectivity, and 0.61–0.70 for alexithymia. In this study, the internal consistency coefficients for the five facet scales ranged from adequate to very good (Cronbach’s α for antagonism = 0.699, 95% CI [0.668–0.728], for impulsivity = 0.797, 95% CI [0.776–0.816], for hedonic capacity = 0.732, 95% CI [0.705–0.758], for negative affectivity = 0.745, 95% CI [0.719–0.769], and for alexithymia = 0.604, 95% CI [0.564–0.642]).

### Data preparation and statistical analyses

Variables in the dataset were initially screened for distribution abnormalities and outliers [111]. Subsequently, the screening for distribution revealed that variables exhibited skewness and kurtosis (for further details, refer to the Kolmogorov–Smirnov test results presented in Table 6 of the supplementary material). Consequently, the assumptions of normality, linearity, and homoscedasticity for the variables were not adequately met [60, 119]. However, the unequal sample size reflected true differences in the numbers of respondents with high gambling severity [14, 42, 114], so artificially equalizing them would have obscured these differences and compromised generalizability [111].

Chi-square testing, Spearman’s correlation testing, one-way ANOVA, and ordinal logistic regression were used in this study. All analyses are robust when dealing with heterogeneous variances, so no other testing was required [62, 89]. Additionally, because the correlation analysis involved multiple simultaneous comparisons, we applied Bonferroni correction to reduce the likelihood of false-positive results [19, 57, 77]. We used the Games–Howell procedure for post hoc tests because it controls the Type I error rate while maintaining statistical power and accuracy under these conditions [40]. Additionally, a Welch’s ANOVA test was conducted as Levene’s test indicated (*p* < 0.001) that the assumption of homogeneity of variance had been violated [55, 64, 82]. While other assumption for the ordinal regression, including the assumption of independent observations, no perfect multicollinearity among independent variables and linearity with the outcome variable—were met [89], the assumption of proportional odds was violated, as the general model fit was inferior to that of the null model. Such occurrences are relatively common in large samples [85]. However, the violation of proportional odds assumption does not undermine our findings, as the robustness of ordinal regression models can accommodate such deviations, especially in large sample sizes such as the present one.

To further ensure the robustness of our findings, we conducted sensitivity analyses employing ordinal logistic regression frameworks based on both the original four-level categorization of the NODS and alternative two- and three-level classifications. This approach enabled us to examine whether the primary results remained consistent across varying operationalizations of gambling severity [98]. The frequencies of the four classification categories, based on the NODS-SA cut-points, were calculated according to instructions and then divided into the following groups: individuals with non-problem gambling (*n* = 850 [81%]), at-risk gambling (*n* = 119 [11%]), problem gambling (*n* = 42 [4%]), and probable gambling disorder (*n* = 42 [4%]). Additionally, the gender variable was adjusted before subsequent analyses, given that one participant specified “other” and two chose “prefer not to say.” The adjustments meant that these three cases were assigned to the male gender based on visually inspecting the output of gender-stratified chi-square and ANOVA analyses. These three cases were excluded from gender comparisons but imputed in all other analyses.

Data were analyzed using IBM SPSS Statistics version 29.0, with the significance level set at *p* < 0.05, except for the correlation analyses, which were Bonferroni adjusted with a significance threshold of *p* < 0.00038.

## Results

### Background characteristics

A significantly larger proportion of men than women reported some form of gambling-related problems (at-risk gambling, problem gambling, probable gambling disorder), with men overrepresented across all categories, comprising between 66.4% and 66.7%, while women accounted for only 33.3% to 33.6%. This stands in contrast to the non-problem gambling group, where the gender distribution was nearly equal, with men representing 52.5% and women 47.5% (*χ*^*2*^[3, *N* = 1053] = 13.04, *p* < 0.001). Beyond gender differences, age also emerged as a distinguishing factor across gambling severity levels. Individuals with probable gambling disorder (*M* age = 33.67 years, *SD* = 13.02) and those experiencing gambling problems were significantly younger (*M* age = 36.62 years, *SD* = 14.34) than those with at-risk gambling (*M* age = 44.01 years, *SD* = 16.25) and non-problem gambling (*M* age = 50.35 years, *SD* = 17.08; *F*2, 100.45 = 32.94, *p* < 0.001, *ωp*^*2*^ = 0.091). In addition, variation in employment status was also observed across gambling severity groups. The proportion of individuals working part or full time was significantly higher among those with at-risk gambling (79.0%), gambling problems (73.8%) and gambling disorder (80.5%) than among those with non-problem gambling (63.7%), representing a significant difference among the four groups (*χ*^2^[3, *N* = 1051] = 15.88, *p* < 0.001). In contrast, there were more retired people among those without gambling problems (25.3%) than among those with at-risk gambling (9.2%), problem gambling (4.7%) and gambling disorder (4.8%; *χ*^2^[3, *N* = 1053] = 31.71, *p* < 0.001). Neither marital status nor income differed significantly between the groups (see Table 1 for more detailed information; regarding age differences, see Table 4).

**Table 1:** Comparisons of background characteristics between gambling severity groups

	Non-problem gambling % (*n* = 850)^a^	At risk gambling % (*n* = 119)^a^	Problem gambling % (*n* = 42)^a^	Gambling disorder % (*n* = 42)^a^	*P*^b^	*ES*^b^
*Gender*						
Male	52.5 (446)	66.4 (79)	66.7 (28)	66.7 (28)	0.05	.111
Female	47.5 (404)	33.6 (40)	33.3 (14)	33.3 (14)		
*Marital status*						
Married/cohabiting without children	27.7 (235)	25.4 (30)	32.6 (14)	23.8 (10)	0.43	.053
Married/cohabiting with children	40.3 (342)	37.7 (41)	25.6 (11)	33.3 (14)		
Single without children	24.7 (210)	32.2 (38)	30.2 (13)	31.0 (13)		
Single with children	7.3 (62)	7.6 (9)	11.6 (5)	11.9 (5)		
*Education*						
Elementary school	8.5 (72)	6.7 (8)	2.3 (1)	9.5 (4)	0.19	.062
High school	33.5 (285)	31.1 (37)	39.5 (17)	40.5 (17)		
Post-secondary education	16.2 (138)	21.0 (25)	18.6 (8)	28.6 (12)		
University	41.8 (356)	41.2 (49)	39.5 (17)	21.5 (9)		
*Occupation*^c^						
Work (full or part time)	63.6 (541)	79.0 (94)	73.8 (31)	80.5 (33)	< 0.01	.123
Study	6.7 (57)	4.2 (5)	14.3 (6)	14.6 (6)	0.03	.090
Job-seeking	1.8 (15)	4.2 (5)	11.9 (5)	4.8 (2)	< 0.01	.134
Retirement pension	25.3 (215)	9.2 (11)	4.7 (2)	4.8 (2)	< 0.01	.174
Sickness benefit	4.0 (34)	5.9 (7)	7.0 (3)	0.0 (0)	0.30	.058
Other	3.1 (26)	4.2 (5)	0.0 (0)	2.4 (1)	0.43	.588
*Monthly income (Euros)*^d^						
< 1333	13.9 (118)	12.7 (15)	11.9 (5)	9.5 (4)	0.36	.064
1333–3110	41.5 (353)	44.1 (52)	47.6 (20)	59.5 (25)		
3111–4444	32.0 (272)	28.0 (33)	31.0 (13)	26.2 (11)		
4445–6666	10.0 (85)	11.9 (14)	2.4 (1)	4.8 (2)		
> 6666	2.7 (23)	3.4 (4)	7.1 (3)	0.0 (0)		
*Ethnic origin*						
Swedish with two native parents	82.5 (701)	68.1 (81)	66.7 (28)	73.2 (30)	< 0.01	.098
Swedish with one foreign parent	7.5 (64)	17.6 (21)	9.5 (4)	9.8 (4)		
Swedish with two foreign parents	3.5 (30)	2.5 (3)	11.9 (5)	4.9 (2)		
Foreign	6.5 (55)	11.8 (14)	11.9 (5)	12.2 (5)		

^a^Percentage (%) and number of participants (*n*)

^b^*p* = statistical significance level and *ES* = Cramer´s V (effect size)

^c^Variables were recoded to account for multiple response options regarding occupation. In cases in which respondents selected both work and study as concurrent activities, the primary employment category of work (including both full- and part-time work) was assigned. This approach ensured a consistent and clear categorization of the respondents’ main employment status

^d^Expressed in the survey in discrete values in SEK, corresponding to approximately EUR 0.09

The percentage and relative frequency of online and offline gambling activities differed significantly (*χ*^2^[6, *N* = 1052] = 60.25, *p* < 0.001), with a higher proportion of people with at-risk gambling (84.9%), problem gambling (97.6%) and gambling disorder (87.8%) more frequently gambling online compared with the non-problem gambling group (60.1%). Significant differences in the frequency of different gambling activities were also found among the gambling groups. The percentage distribution indicates that those experiencing at-risk gambling, problem gambling and gambling disorder seem to engage more frequently in different types of gambling activities than do individuals without gambling problems, with casino games (36.6%), sports betting (31.7%), and number games (23.8%) being the most commonly observed (for further details, see Table 2).

**Table 2:** Comparisons of gambling activities in gambling severity groups

	Non-problem gambling % (*n* = 850)^a^	At risk gambling % (*n* = 119)^a^	Problem gambling % (*n* = 42)^a^	Gambling disorder % (*n* = 42)^a^	*P*^b^	*ES*^b^
*Form and frequency of gambling activities*^c^						
Online	60.1 (511)	84.9 (101)	97.6 (41)	87.8 (36)	< 0.01	.169
Offline	30.4 (258)	13.4 (16)	2.4 (1)	12.2 (5)		
Other	9.5 (81)	1.7 (2)	0.0 (0)	0.0 (0)		
*Lotterie*^c^						
Not gambled	18.2 (155)	17.6 (21)	23.8 (10)	14.6 (6)	0.83	.040
Last year	39.3 (334)	39.5 (47)	42.9 (18)	43.9 (18)		
Last month	30.1 (256)	28.6 (34)	21.4 (9)	22.0 (9)		
Last week or more	12.4 (105)	14.3 (17)	11.9 (5)	19.5 (8)		
*Number games*^c^						
Not gambled	57.6 (490)	50 (59)	57.1 (24)	31.0 (13)	0.05	.073
Last year	14.7 (125)	19.5 (23)	21.4 (9)	26.2 (11)		
Last month	10.7 (91)	(17)	9.5 (4)	19.0 (8)		
Last week or more	16.9 (144)	16.1 (19)	11.9 (5)	23.8 (10)		
*Sports betting*^c^						
Not gambled	66.8 (568)	40.8 (49)	28.6 (12)	19.5 (8)	< 0.01	.186
Last year	16.9 (144)	(28)	14.3 (6)	31.7 (13)		
Last month	9.4 (80)	(22)	26.2 (11)	17.1 (7)		
Last week or more	6.8 (58)	17.5 (21)	31.0 (13)	31.7 (13)		
*Horse betting*^c^						
Not gambled	60.6 (515)	56.7 (68)	54.8 (23)	31.0 (13)	0.05	.073
Last year	(146)	20.0 (24)	19.0 (8)	33.3 (14)		
Last month	10.9 (93)	12.5 (15)	14.3 (6)	14.3 (6)		
Last week or more	11.3 (96)	10.8 (13)	11.9 (5)	21.4 (9)		
*Poker games*^c^						
Not gambled	91.4 (777)	74.2 (89)	59.5 (25)	38.1 (16)	< 0.01	.231
Last year	(57)	15.0 (18)	16.7 (7)	35.7 (15)		
Last month	(9)	10.0 (12)	16.7 (7)	19.0 (8)		
Last week or more	0.8 (7)	0.8 (1)	7.1 (3)	7.1 (3)		
*Casino games*^c^						
Not gambled	88.6 (753)	57.1 (68)	40.5 (17)	14.6 (6)	< 0.01	.308
Last year	(66)	21.0 (25)	19.0 (8)	26.8 (11)		
Last month	2.2 (19)	14.3 (17)	(9)	22.0 (9)		
Last week or more	1.4 (12)	7.6 (9)	19.0 (8)	36.6 (15)		
*Slot machine*^c^						
Not gambled	92 (787)	71.4 (85)	52.4 (22)	33.3 (14)	< 0.01	.261
Last year	6.5 (55)	(22)	28.6 (12)	35.7 (15)		
Last month	(10)	6.7 (8)	7.1 (3)	14.3 (6)		
Last week or more	0.4 (3)	3.4 (4)	11.9 (5)	16.7 (7)		
*Bingo games*^c^						
Not gambled	67.3 (573)	60.5 (72)	46.5 (20)	35.7 (15)	< 0.01	.124
Last year	26.8 (228)	(36)	34.9 (15)	35.7 (15)		
Last month	(33)	7.6 (9)	11.6 (5)	16.7 (7)		
Last week or more	2 (17)	1.7 (2)	7.0 (3)	11.9 (5)		
*Betting*^c^						
Not gambled	93.5 (795)	78.2 (93)	65.1 (28)	39.0 (16)	< 0.01	.245
Last year	(38)	7.6 (9)	11.6 (5)	34.1 (14)		
Last month	1.4 (12)	10.1 (12)	18.6 (8)	12.2 (5)		
Last week or more	0.6 (5)	4.2 (5)	4.7 (2)	14.6 (6)		

^a^Percentage (%) of number of participants and number of participants (*n*)

^b^*p* = statistical significance level and *ES* = Cramer´s V (effect size)

^c^Frequency of engagement in this gambling activity: never, in the last year, in the last month, or in the last week or more recently

### Associations among craving, gambling problems, mental health symptoms, affects, personality, age, and gender

Spearman correlation analysis showed that all three craving sub-scales were strongly positively associated with gambling problem severity (desire: *r*_*s*_ = 0.56, *p* < 0.003; anticipation: *r*_*s*_ = 0.44, *p* < 0.003; and relief: *r*_*s*_ = 0.50, *p* < 0.001) and weakly to moderately associated with symptoms of anxiety (*r*_s_ = 0.26–0.31, *p* < 0.003), symptoms of depression (*r*_s_ = 0.26–0.28, *p* < 0.003), negative affect (*r*_s_ = 0.26–0.33, *p* < 0.003), and the personality traits antagonism (*r*_s_ = 0.19–0.24, *p* < 0.003), impulsivity (*r*_s_ = 0.17–0.19, *p* < 0.003), negative affectivity (*r*_s_ = 0.21–0.22, *p* < 0.003), and alexithymia (*r*_s_ = 0.06–0.14, *p* < 0.05). Conversely, the craving subscales exhibited weak to moderately negative associations with positive affect (*r*_s_ = 0.12–0.14, *p* < 0.003), the personality trait hedonic capacity (*r*_s_ = 0.14–0.18*, p* < 0.001), age (*r*_s_ = –0.30, *p* < 0.003), and gender (*r*_s_ = –0.11–0.16, *p* < 0.003) (for more detailed information, see Table 3).

**Table 3:** Spearman’s correlation coefficient for associations among gambling problems, craving, mental health symptoms, affects, personality traits, age, and gender (*N* = 1053)

	1	2	3	4	5	6	7	8	9	10	11	12	13	14
1. **NODS-SA Last year**	-													
2. **GACS-Desire**	.562***	**-**												
3. **GACS-Anticipation**	.442***	.598***	**-**											
4.** GACS-Relief**	.502***	.652***	.518***	**-**										
5.** HADS-Anxiety**	.330***	.311***	.275***	.263***	**-**									
6. **HADS-Depression**	.307***	.288***	.258***	.268***	.723***	**-**								
7.** PANAS-Positive affect**	–.109***	–.129***	–.147***	–.134***	–.362***	–.508***	**-**							
8.** PANAS-Negative affect**	.326***	.339***	.282***	.264***	.787***	.627***	–.253***	**-**						
9. **HP5I-Antagonism**	.229***	.246***	.222***	.191***	.346***	.316***	–.121***	.371***	**-**					
10. **HP5I-Impulsivity**	.264***	.192***	.172***	.187***	.386***	.326***	–.080**	.370***	.391***	**-**				
11.** HP5I-Hedonic capacity**	–.191***	–.187***	–.175***	–.148***	–.412***	–.542***	.648***	–.394***	–.205***	–.141***	**-**			
12. **HP5I-Negative affectivity**	.241***	.224***	.212***	.228***	.672***	.540***	–.333***	.656***	.333***	.406***	–.360***	**-**		
13. **HP5I-Alexithymia**	.122***	.147***	.066*	.150***	.038	.165***	–.115***	.075*	.301***	.154***	–.162***	.098***	**-**	
14.** Age**	–.243***	–.302***	–.306***	–.300***	–.381*	–.301***	.076*	–.440***	–.144***	–.183***	.147***	–.289***	–.002	
15.** Gender**	–.106***	–.162***	–.168***	–.116***	.095***	**.009**	.008	.045	–.190***	.045	.088**	.163***	–.275***	.029

Bold values represent significant correlation coefficients: * *p* < 0.05, ** *p* < 0.01, *** *p* < 0.0038; significant correlation coefficients are Bonferroni corrected

NODS-SA Last year: National Opinion Research Center DSM-IV Screen for Gambling Problems; GACS-Desire: strong, urgent desire to gamble; GACS-Anticipation: intention to gamble that was anticipated to be fun and enjoyable; GACS-Relief: relief from negative experiences expected from gambling; HADS-Anxiety: assessing symptom severity of anxiety; HADS-Depression: assessing symptom severity of depression; PANAS-Positive affect: propensity to experience positive emotions; PANAS-Negative affect: propensity to experience negative emotions; HP5I-Antagonism: hostile behavior; HP5I-Impulsivity: impulsive behavior; HP5I-Hedonic capacity: capacity to experience pleasure; HP5I-Negative affectivity: experience of negative feelings; HP5I-Alexithymia: inability to verbally express emotions. Age (18–84 years of age). Gender (female/male, reference category female)

### Differences in levels of craving and age among gambling severity groups

Table 4 presents the results of the one-way ANOVAs for differences among gambling severity groups in the craving subscales and age.

**Table 4:** Variance analysis results: differences in levels of craving and age among gambling severity groups^a^

	Non-problem gambling (*n* = 850)^b^	At-risk gambling (*n* = 119)^b^	Problem gambling (*n* = 42)^b^	Gambling disorder (*n* = 42)^b^	*F*-value (df1, df2)^d^	*P*^e^	*ES*^e^
	Mean ± *SD*^c^	Mean ± *SD*^c^	Mean ± *SD*^c^	Mean ± *SD*^c^			
GACS-Desire	1.18 ± 0.53	2.04 ± 1.14	3.07 ± 1.49	3.84 ± 1.44	87.65 (3, 89.15)	<.001**	.475
GACS-Anticipation	2.44 ± 1.22	3.85 ± 1.39	4.67 ± 1.27	4.41 ± 1.30	96.17 (3, 95.79)	<.001**	.271
GACS-Relief	1.32 ± 0.81	2.08 ± 1.15	3.17 ± 1.58	3.62 ± 1.45	65.22 (3, 91.12)	<.001**	.324
Age	50.35 ± 17.08	44.01 ± 16.25	36.62 ± 14.34	33.67 ± 13.02	32.94 (3, 100.45)	<.001**	.091

^a^Welch one-way ANOVA comparisons among gambling severity groups for: NODS-SA Last year, a DSM-IV screen for gambling problem severity; GACS, a screen for levels of craving desire, anticipation, and relief; and age (18–84 years)

^b^*n* = number of participants in each gambling severity group

^c^Mean ± *SD* = mean value and standard deviation

^d^*F*-value and df = degree of freedom

^e^Statistical significance level: * *p* < 0.05 and ** *p* < 0.01; *ES* = ω^2^ (omega-squared effect size)

The one-way ANOVA executed on the craving subscales showed that gambling groups differed significantly in levels of desire (*F*2, 135.19 = 123.36, *p* < 0.001; *ωp*^*2*^ = 0.475), anticipation (*F*2, 153.72 = 145.05, *p* < 0.001, *ωp*^*2*^ = 0.271), and relief (*F*2, 141.48 = 95.26, *p* < 0.001, *ωp*^*2*^ = 0.324), with a significant effect in multiple comparisons on all subscales among the groups (all *p* values < 0.01). People with problem gambling and gambling disorder reported higher levels of craving in all craving subscales than did the at-risk gambling or non-problem gambling group. Notably, this pattern was consistent across all dimensions of craving, with the exception of the anticipation subscale, wherein individuals classified with problem gambling demonstrated marginally higher levels of craving than those meeting criteria for gambling disorder. Nevertheless, despite this exception, findings indicate a clear gradient, with craving intensity increasing in line with gambling severity. Table 7 in the supplementary material provides a detailed summary of the multiple comparison test results across the different gambling severity groups.

### Craving’s association with gambling problems when controlling for mental health symptoms, affects, personality traits, gender, and age

**Fig. 1:**
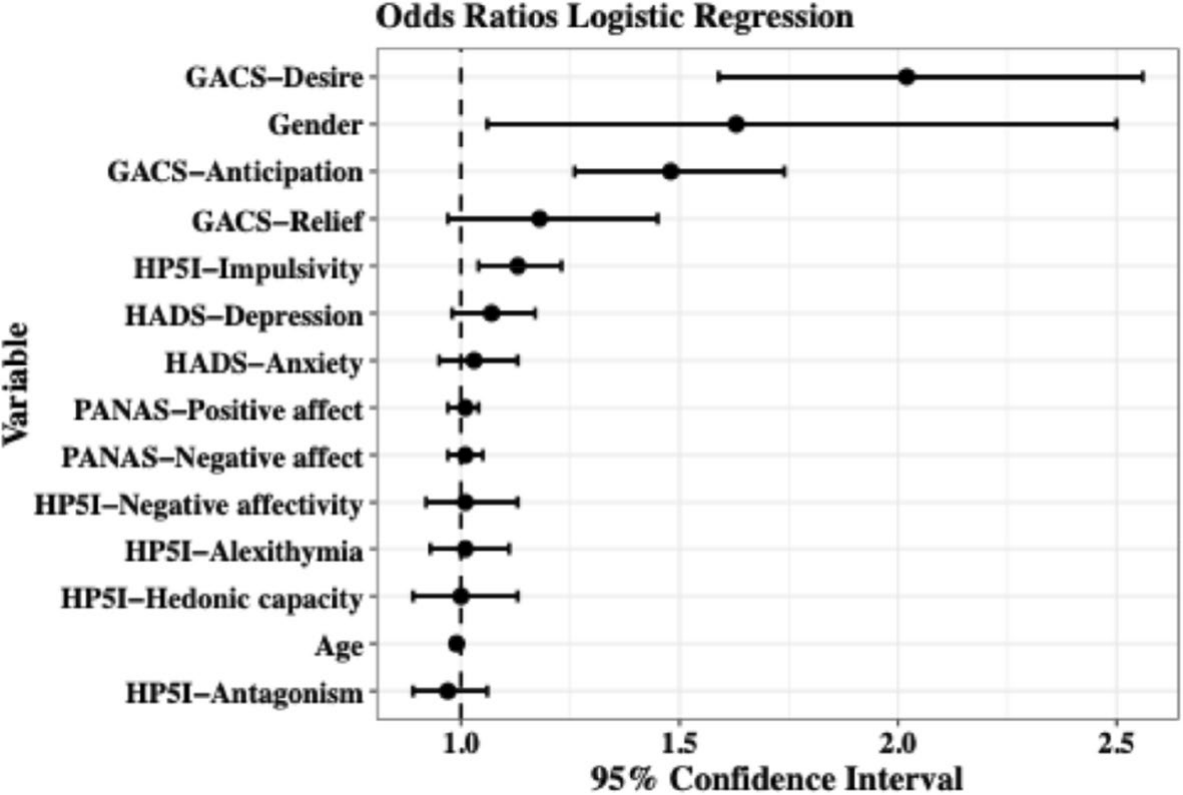
Note: Forest plot displaying odds ratios and 95% confidence intervals from the ordinal regression model with gambling severity as the outcome variable (i.e., Non-problem gambling, At-risk gambling, Problem gambling, and Gambling disorder), and all variables displayed in the plot as predictors. Closed circles represent point estimated odds ratios, and whiskers represent 95% confidence intervals; variables are displayed in descending order based on the magnitude of the odds ratios. For detailed estimates of coefficients and effects, see Table 5

In the univariate logistic regression analyses (Table 5), all predictors showed statistically significant associations with gambling problem severity, and the 95% confidence intervals (CI) for all odds ratios (OR) did not include 1.0. These findings indicated that each variable, when examined independently, was reliably related to increased odds of gambling problems. To determine whether these associations held when accounting for the shared variance among predictors, we next estimated a multivariable ordinal logistic regression model including all factors simultaneously. In this multivariable model, based on the original four-level classification derived from the NODS-SA cut-points, we found that the craving dimensions desire (OR = 2.02, CI [1.59–2.56], *p* < 0.001) and anticipation (OR = 1.48, CI [1.26–1.74], *p* < 0.001) remained significantly associated with increased odds of experiencing gambling problems (NODS ≥ 1), even when controlling for all other variables included in the model. In addition, the personality trait impulsivity increased the odds of engaging in problematic gambling behavior by 1.13 times (CI [1.04–1.23], *p* < 0.004), while being male increased the odds by 1.63 times (CI [1.06–2.50], *p* < 0.024). Notably, although no other variables were significant, craving dimension relief exhibited tendencies toward increasing the odds of experiencing gambling problems (OR = 1.18, CI [0.97–1.45], *p* < 0.096) (see Table 5 and Fig. 1 for further details).

**Table 5:** Results of logistic regression analysis of whether craving predicts problematic gambling (Non-problem gambling *n* = 850 vs. At-risk gambling *n* = 119, Problem gambling *n* = 42, and Gambling disorder *n* = 42) when controlling for additional risk factors

	Univariate Model^a^			Multivariable Model^b^			
	OR^c^	CI 95% for OR^c^		OR^c^	CI 95% for OR^c^		
		Lower	Upper		Lower	Upper	
GACS-Desire	3.77**	3.20	4.43	2.02**	1.59	2.56	
GACS-Anticipation	2.45**	2.16	2.78	1.48**	1.26	1.74	
GACS-Relief	2.56**	2.25	2.92	1.18	0.97	1.45	
HADS-Anxiety	1.23**	1.18	1.28	1.03	0.95	1.13	
HADS-Depression	1.28**	1.22	1.34	1.07	0.98	1.17	
PANAS-Positive affect	0.96**	0.94	0.98	1.01	0.97	1.04	
PANAS-Negative affect	1.12**	1.10	1.14	1.01	0.97	1.05	
HP5I-Antagonism	1.27**	1.19	1.35	0.97	0.89	1.06	
HP5I-Impulsivity	1.32**	1.24	1.40	1.13**	1.04	1.23	
HP5I-Hedonic capacity	0.79**	0.73	0.85	1.00	0.89	1.13	
HP5I-Negative affectivity	1.26**	1.19	1.34	1.01	0.92	1.13	
HP5I-Alexithymia	1.15**	1.07	1.23	1.01	0.93	1.11	
Age	0.96**	0.95	0.97	0.99	0.98	1.00	
Gender^d^	1.75**	1.27	2.41	1.63*	1.06	2.50	

Statistical significance level: * *p* < 0.05 and ** *p* < 0.01

^a^Univariate model: Ordinal logistic regression analysis, controlling for each independent variable’s individual influence on the outcome variable

^b^Multivariable model: Ordinal logistic regression with all predictors were entered simultaneously to estimate their unique contributions to gambling problem severity

^c^OR = odds ratio; CI 95% for OR = 95% confidence interval for odds ratio

^d^Reference category female

Furthermore, the Nagelkerke *R*^*2*^ value of 0.46 indicated that the included predictors jointly accounted for a substantial proportion of the variance in the outcome variable, distinguishing individuals across the full spectrum of gambling severity—from non-problem gambling through at-risk and problem gambling to probable gambling disorder. The model significantly outperformed the baseline in predicting gambling problem status [*χ*^*2*^(14) = 427.637, *p* < 0.001], and the consistency of these results across the alternative categorizations in the sensitivity analyses further supports the robustness of these findings. Detailed comparable statistical results are presented in Table 8 in the supplementary material.

## Discussion

The first objective of this study was to examine whether craving was related to gambling severity, mental health symptoms, affects, and personality traits.

We found that all three craving subscales were strongly associated with gambling severity, and that there were differences among those with probable gambling disorder, gambling problems, with at-risk gambling, and without gambling problems. Our findings showed that those with gambling disorder, gambling problems and at-risk gambling displayed significantly higher levels of anticipation (intention to gamble that was anticipated to be fun and enjoyable), desire (strong, urgent desire to gamble), and relief (relief from negative experiences expected from gambling) then did those in the non-problem category. The current findings are consistent with most previous research [10, 25, 26, 37], although two studies have somewhat discrepant findings [12, 15]. The strong associations and substantial effect sizes suggest that there may be clinically meaningful differences in craving experiences across risk domains. This lends support that craving could, in part, be a central driving factor underlying the escalation of problematic gambling behavior.

A growing body of literature emphasize craving as a critical factor in the development and maintenance of gambling disorder. In fact, multiple experts consider that craving is an essential feature in gambling disorder that is reflected in aspects such as loss of control, chasing losses, and persistent gambling despite negative consequences (e.g., [7, 29, 103, 115]). Neurobiological evidence reinforces these associations with increased activity in the insula and ventral striatum [66], mirroring patterns observed in substance use disorders. Additionally, preparatory rituals and anticipatory imagery reported by individuals with gambling disorder resemble the “incentive salience” and dopaminergic anticipatory responses found in substance craving, suggesting a shared underlying mechanism [54, 99, 100]. Given these converging lines, and reflecting its clinical relevance and predictive value, several researchers have advocated for the inclusion of craving as a diagnostic criterion for gambling disorder (e.g., [4, 54, 73, 97]), similar to its established role in the DSM-5 for substance use disorders [3, 104].

Taken together, our findings add to the converging empirical, clinical, and neurobiological evidence by demonstrating that craving is highly prevalent among individuals across the spectrum of gambling severity, and therefore appears to serves as a central motivational force driving gambling behavior. This further supports the proposals to include craving as a diagnostic criterion in future revisions of the DSM, as it could enhance the diagnostic validity of gambling disorder and improve early identification of individuals at clinical risk, much as it has in the case of substance use disorder.

Moderate associations were also observed between the craving subscales, and symptoms of anxiety and depression. This is partly consistent with previous research, which has highlighted that people with gambling problems often exhibit co-occurring mood and anxiety disorders [9, 81, 110] and frequently engage in gambling as a way to ameliorate mood (e.g., [86, 96, 116]). However, two more recent studies examining the associations among the craving subscales, depression, and anxiety [22, 24] did not identify any significant relationships. To the best of our knowledge, only one prior study has found depression—but not anxiety—to be a significant factor influencing the craving subscales of desire and relief [125]. It is therefore noteworthy that the present study’s results demonstrate that both anxiety and depression are moderately associated with all three craving subscales.

We also found moderately positive associations between all three craving subscales and negative affects (reflecting a heightened tendency to experience negative emotions), alongside a small but significant negative association between the craving subscales and positive affects (indicating a reduced tendency to experience positive emotions). This is perhaps unsurprising, as the relationship among high negative affects, low positive affects, and elevated levels of anxiety and depression has consistently been documented in the literature (e.g., [35, 124]). Young and Wohl [125] reported generally weaker associations between some of the craving subscales and both negative affects and positive affects; however, they examined a sample with different characteristics, which may be one explanation of the dissimilar findings.

As gamblers with symptoms of anxiety, depression, and negative affect often engage in gambling as a means of mood regulation, this study’s associations might be understood through the lens of most recent models of gambling problems, which place emotional regulation (ER) difficulties in a dominant position in their etiological and maintenance pathways [86, 96, 116]. However, the present study also provides new insights by emphasizing the role of craving in these ER difficulties, which often stem from underlying anxious and depressive symptoms found in individuals experiencing gambling problems [9, 44, 58, 92]. Based on these perspectives, distress reduction and escape from unwanted emotions may dominate the strategy selection process, with craving driving a narrow and rigid escape/avoidance strategy and limiting the ability to choose adaptive, context-appropriate coping options [87, 90, 96]. Craving may thereby lower emotional clarity and awareness, fostering a bias toward immediate rewards and reinforcing maladaptive gambling behaviors [87].

It may be contradictory that the craving subscale anticipation, which is associated with positive reinforcement, was also associated with depression, anxiety, and negative affects [36, 120]. These seemingly contradictory motives—such as a strong desire to gamble driven by anticipated enjoyment and positive reinforcement (e.g., desire and anticipation), alongside the urge to escape or avoid negative experiences through gambling (e.g., relief)—may therefore coexist within the same individual or manifest differently across various groups of gamblers (e.g., [74, 87, 88]). Ultimately, craving seems to function as a contributing mechanism in suppressing aversive emotional states stemming from an increased propensity for negative affects and a diminished capacity for positive affects.

Nevertheless, based on these findings, it remains unclear whether the associations among the craving subscales and emotional vulnerabilities, as well as the comorbidity of depression and anxiety symptoms, are consequences rather than factors contributing to problematic gambling behavior (excessive gambling behavior).

In this study we also identified significant associations between craving and personality traits. This partly aligns with previous research, which has consistently reported links between maladaptive personality traits and gambling severity (e.g., [31, 38, 65, 108]). Both antagonism (referring to aspects of a hostile interpersonal style or expressive hostility) and impulsivity (referring to choosing rapidly with little thought and a non-planning tendency) were positively associated with the craving subscales, which is in line with a recent study that identified associations among the three craving subscales, antagonism, and disinhibition (a facet related to impulsivity) [25]. This confirms the tendency to act impulsively with non-planning-oriented behavior and to chase losses, along with a propensity for immediate gratification often observed among individuals experiencing gambling disorder [39, 56, 121]. In a clinical context, the antagonism trait may also indicate a propensity for more asocial behavior, as seen in those with pathological gambling problems [11, 63, 91, 107].

The craving subscales were negatively associated with hedonic capacity, which is to some extent consistent with previous studies. For instance, Ciccarelli et al. [25] identified associations between detachment (a facet inversely related to hedonic capacity) and the craving subscales desire and relief. Similarly, Tavares et al. [112] found that reward dependence, a facet related to hedonic capacity, was associated with craving as a unidimensional construct. This suggests that individuals with a lower propensity to experience joy or happiness may be vulnerable to craving and thereby more likely to miss gambling during abstinence. The three craving subscales were also associated with the personality trait negative affectivity (a facet of neuroticism and representing nervous tension and distress) along with alexithymia (e.g., a facet related to psychoticism and characterized by a lack of interest in recognizing and understanding feelings). The literature has widely demonstrated a strong relationship among alexithymia, negative affectivity, and gambling severity (e.g., [70, 75, 108, 109]), but has not previously shown associations with craving subscales [25].

Taken together, this implies that lack of emotional competence, clarity, and awareness [116, 122] may drive both craving and gambling, as individuals with gambling problems apparently turn to gambling as a way to cope with negative emotional states and to enhance mood [68, 87, 90]. Because these findings are merely correlational, they do not provide evidence that the traits necessarily cause craving or problem gambling, rather, they are consistent with possible underlying causes of problem gambling. Nonetheless, collectively, this supports the theory that personality factors may have implications for the etiology of craving and problem gambling.

Regarding our second objective, to our knowledge this is the first study to simultaneously explore whether different aspects of craving are related to the severity of gambling problems, when controlling for mental health symptoms, affects, personality traits, age, and gender in adults who gamble. We found that different aspects of craving were indeed related to the severity of gambling problems, even when controlling for the above-mentioned variables. More specifically, the findings showed that the craving subscale desire (a strong, urgent desire to gamble) and the craving subscale anticipation (gambling anticipated to be fun and enjoyable) predicted problematic gambling behavior. While these results support the findings of Ciccarelli et al. [25] and Cosenza et al. [28], they only partly align with those of Battaglia et al. [10]. In addition to desire and anticipation, Battaglia et al. [10] found that the relief subscale (representing the expected relief from negative experiences obtained through gambling) also predicted problematic gambling. It was therefore somewhat surprising that only the playful and positive aspects of craving appeared to be involved in gambling problems, rather than the emotional relief from negative affects that has been observed among adult individuals with gambling disorder [32, 82, 87].

The divergent findings may stem from the fact that, among non-clinical gamblers like those in our and Ciccarelli et al.'s [25] samples, the most commonly reported trigger is positive affects (see also [27, 28]), in contrast to gamblers with clinical disorders, such as those examined by Battaglia et al. [10], in whom negative moods are more common (see also [74]). This could explain the emergence of positive over negative aspects of craving, as it has been suggested that the relative weights of the positively and negatively valanced components of craving could change as the addictive process progresses, and problems become more severe [37, 68, 76].

One might speculate that this reflects the highly reinforcing nature of anticipation—i.e., for some, the phase of the strong urgent desire, and anticipating gambling becomes a central component of the craving experience [5], and that distorted expectancy might peak during this phase, when the actual effects remain unknown [67]. In accordance with the above-mentioned anticipatory dopamine response without intake of substances and the reward prediction error, has been suggested to be a common feature across addictions [54]. This could be viewed such that gambling craving might to a larger extent be driven by positive expectation, or anticipated rewards. Although relief cravings have been documented in gambling disorder and are included in measures such as the Gambling Craving Scale [10, 125], they might be more prominent in clinical samples with gambling disorder [74]. Some researchers have even argued that gambling cravings arise not from a desire to escape negative emotions, but rather from a lack of positive experiences [33].

In addition, apart from craving—desire and craving—anticipation, impulsivity and being male also emerged as significant predictors of problematic gambling. Building on this, prior research has consistently demonstrated that men are more likely than women to engage in gambling activities [8, 14, 121], and that being male is a reliable predictor of gambling-related problems [1, 41, 84, 113]. Even though Mestre-Bach et al. [79] did not find that male gender or impulsivity predicted gambling severity (also see [80]), most studies in the field support our finding that both being male and impulsivity are associated with, and serve as reliable predictors of, gambling-related problems (e.g., [20, 61, 93]).

Seen separately, the impulsivity trait has exhibited a robust association with problematic gambling [38, 72]. In particular, various trait measures of impulsivity have been shown to differentiate individuals with gambling problems from control groups and those without gambling problems [23, 46, 56, 71]. Previous research has also indicated that impulsivity has a direct effect on problem gambling [39], with lack of awareness and clarity, which may induce a bias toward immediate rewards and favor the selection of impulsive action (i.e., continuing to gamble for positive reinforcement), consistently exhibiting positive associations with gambling severity [87, 95].

Taken together, male gender, impulsive behavior, and strong anticipation and desire (i.e., the craving subscales anticipation and desire) predict pathological gambling. This finding seems to imply that impulsivity and the playful and positive aspects of craving, such as desire and anticipation, can coexist as predictors of problematic gambling [17, 25], but also, given the effect size, that craving components probably play a more central role, at least in this sample of adults who engage in gambling. This effect is congruent with the proposal that craving consists in part of appetitive or affectively positive components, and that desire and anticipation influence the emergence or regulation of such components [25, 27, 28]. In other words, males with high impulsivity and a tendency to lose control during positive affective states may experience more intense craving, in turn interfering with their attempts to regulate potentially addictive behavior [18, 45, 68].

### Limitations

This study, aside from its strong properties, including the large sample, has some limitations that should be considered when interpreting the findings. First, although the study provides valuable insights, its cross-sectional design does not allow for causal inferences regarding the observed relationships. Second, the use of a online survey for data collection administered to members of a voluntary web panel, may pose risks of both recall bias and selection bias. Individuals who choose to join such panels may also differ systematically from the general population, limiting representativeness. In addition, self-report measures are inherently vulnerable to social desirability bias and inaccuracies in participants’ retrospective reporting.

Although the present results were derived from a population of individuals who gamble, their generalizability is limited to Swedish adult non-clinical individuals who engage in gambling. There is a great need for more studies of craving and problem gambling in other countries, focusing on both non-clinical and clinical individuals who gamble, in order to better elucidate the role of craving, which may manifest itself in different ways. For instance, further research on individuals with gambling disorder and/or research that considers gambling preferences is warranted to extend the present findings. In this regard, researchers could disentangle which craving triggers are more frequently related to dysfunctional gambling habits.

Moreover, while this study offers important new insights, it lacks the depth that could be gained from qualitative material obtained through in-depth interviews or focus groups. The role of subjectively reported craving in gambling addiction treatment and research has yet to be fully understood. Future research should therefore examine how modes of thought relate to relapses processes and treatment trajectories, ideally by following individuals suffering from gambling disorder and track how craving unfolds over time.

Lastly, it is important for future studies to determine the robustness of the observed and non-observed interactions through replication, possibly assessing whether the connections of craving with problem gambling and other risk factors are reliant on factors not considered here, such as gambling modality, relapse, and treatment retention. In this regard, researchers should prioritize the collection of diverse samples allowing for sufficient variability and the use of different methods in order to detect and examine the roles of these factors.

## Conclusion

This study is the first to examine the role of craving in gambling severity, while simultaneously controlling for mental health symptoms, affects, personality traits, age, and gender in gambling adults. The findings highlight a clear association between craving and gambling severity, mental health symptoms, affects, maladaptive personality traits, age, and gender, reinforcing the centrality and sensitivity of craving to the constructs of interest. This remained true even after controlling for the above-mentioned variables, as craving, characterized by a strong and urgent desire to gamble (desire), which is anticipated as being fun and enjoyable (anticipation), alongside impulsivity and being male, still predicted problematic gambling behavior. These associations were robust, positive, and significant across the analyses under scrutiny, supporting previous findings that craving components probably play a central role in problematic gambling behavior. The predominance of the positive aspects of craving highlighted in several studies reinforces the idea that desire and anticipation, along with impulsivity, act as “Trojan horses” in addictive processes for non-clinical individuals who engage in gambling. Despite the research limitations, and although the present findings are unlikely to be the sole risk factors affecting problematic gambling behavior, they make an important contribution and imply that adjusted prevention and treatment approaches could beneficially address such factors, and further reinforce the evidence for considering craving as a potential diagnostic criterion in future revisions of the diagnostic criteria for gambling disorder.

## Supplementary Information

Supplementary Material 1.

## Data Availability

All data generated and/or analyzed during the current study are not publicly available but are available from the corresponding author on reasonable request.
