# Supplementary material for: The role of craving in gambling behavior: examining its relevance and links to psychological distress, personality, and demographics
Source: BMC Psychol. 2025 Dec 12;14:74. doi: 10.1186/s40359-025-03816-4 (PMC12810000; doi:10.1186/s40359-025-03816-4)
Supplement: Supplementary file 1 — Supplementary Material 1. [file 40359_2025_3816_MOESM1_ESM.docx]

**Supplementary material**

**Table 6** Screening for distribution among the variables in the study: Kolmogorov–Smirnov test results for skewness and kurtosis **^a^**

|  | Skewness | *SE* **^b^** | Kurtosis | *SE* **^b^** | Statistic |
| --- | --- | --- | --- | --- | --- |
| **NODS-SA Last year** | 3.53 | .075 | 13.12 | .151 | .449 |
| **GACS-Desire** | 2.66 | .075 | 7.30 | .151 | .373 |
| **GACS-Anticipation** | 0.54 | .075 | –0.24 | .151 | .107 |
| **GACS-Relief** | 2.08 | .075 | 3.69 | .151 | .379 |
| **HADS-Anxiety** | 0.97 | .075 | 0.36 | .151 | .149 |
| **HADS-Depression** | 1.55 | .075 | 2.41 | .151 | .220 |
| **PANAS-Positive affect** | –0.41 | .075 | –0.02 | .151 | .072 |
| **PANAS-Negative affect** | 1.30 | .075 | 1.33 | .151 | .162 |
| **HP5I-Antagonism** | 0.33 | .075 | –0.10 | .151 | .104 |
| **HP5I-Impulsivity** | 0.44 | .075 | –.029 | .151 | .110 |
| **HP5I-Hedonic capacity** | –0.59 | .075 | 0.81 | .151 | .177 |
| **HP5I-Negative affectivity** | 0.58 | .075 | –0.37 | .151 | .133 |
| **HP5I-Alexithymia** | 0.20 | .075 | –0.39 | .151 | .094 |
| ^a^ Kolmogorov–Smirnov test results for skewness and kurtosis in: NODS-SA Last year, gambling problem severity; GACS, levels of craving desire, anticipation, and relief; HADS, symptoms of anxiety and depression; PANAS, propensity to experience positive and negative emotions; and HP5I, health-relevant personality traits  ^b^ *SE* = standard error | | | | | |

**Table 7**  Games–Howell post hoc test for multiple comparisons among gambling severity groups **^a^**

|  | **^0^** Non-problem gambling |  | **^1^** At-risk gambling |  | **^2^** Problem gambling |  | **^3^** Gambling disorder |  | | |
| --- | --- | --- | --- | --- | --- | --- | --- | --- | --- | --- |
|  | Mean difference (I–J) **^c^** | *P* **^d^** | Mean difference (I–J) **^c^** | *P* **^d^** | Mean difference (I–J) **^c^** | *P* **^d^** | Mean difference (I–J) **^c^** | *P* **^d^** | | |
| ***n* ^b^** | 850 |  | 119 |  | 42 |  | 42 |  | | |
| **GACS-Desire** | 1: –0.859  2: –1.885  3: –2.660 | <.001**  <.001**  <.001** | 0: 0.859  2: –1.025  3: –1.800 | <.001**  <.001**  <.001** | 0: 1.885  1: 1.025  3: –0.774 | <.001**  <.001**  <.001** | 0: 2.660  1: –1.800  2: 0.774 | <.001**  <.001**  .082 | | |
| **GACS-Anticipation** | 1: –1.415  2: –2.235  3: –1.973 | <.001**  <.001**  <.001** | 0: 1.415  2: –0.819  3: –0.557 | <.001**  <.004*  .100 | 0: 2.235  1: 0.819  3: 0.261 | <.001**  <.004*  .790 | 0: 1.973  1: 0.557  2: –0.261 | <.001**  .100  .790 | | |
| **GACS-Relief** | 1: –0.759  2: –1.843  3: –2.293 | <.001**  <.001**  <.001** | 0: 0.759  2: –1.084  3: –1.534 | <.001**  <.001**  <.001** | 0: 0.244  1: 0.264  3: 0.330 | <.001**  <.001**  <.001** | 0: 2.293  1: 1.534  2: 0.450 | <.001**  <.001**  .527 | | |
| **Age** | 1: 6.336  2: 13.732  3: 16.677 | <.001**  <.001**  <.001** | 0: –6.336  2: 7.395  3: 10.340 | <.001**  .033*  <.001** | 0: –13.732  1: –7.395  3: 2.944 | <.001**  .033*  .758 | 0: –16.677  1: –10.340  2: –2.944 | <.001**  <.001**  .758 | | |
| ^a^ Games–Howell post hoc test for multiple comparisons among gambling severity groups for: NODS-SA Last year, a DSM-IV screen for gambling problem severity; GACS, a screen for levels of craving desire, anticipation, and relief; and age (18–84 years)  ^b^ *n* = number of participants in each gambling severity group  ^c^ I–J = mean differences between groups, with 0 representing Non-problem gambling, 1 representing At-risk gambling, 2 representing Problem gambling, 3 representing Gambling disorder  ^d^ Statistical significance level: * *p* < 0.05 and ** *p* < 0.01 | | | | | | | | |  |  |

| **Table 8** Sensitivity analysis results of Bivariate and Ordinal logistic regression models (two- and three-level classifications of NODS) of whether craving predicts problematic gambling when controlling for other risk factors | | | | | | | |
| --- | --- | --- | --- | --- | --- | --- | --- |
|  | Model **^a^** |  | | Model **^b^** |  | | |
|  | OR **^c^** | CI 95% for OR **^c^** | | OR **^c^** | CI 95% for OR **^c^** | | |
|  |  | Lower | Upper |  | Lower | Upper | |
| **GACS-Desire** | 2.21** | 1.66 | 2.94 | 2.11** | 1.64 | 2.71 | |
| **GACS-Anticipation** | 1.58** | 1.32 | 1.89 | 1.51** | 1.28 | 1.78 | |
| **GACS-Relief** | 1.13 | 0.90 | 1.42 | 1.20 | 0.98 | 1.48 | |
| **HADS-Anxiety** | 1.09 | 0.99 | 1.20 | 1.07 | 0.98 | 1.17 | |
| **HADS-Depression** | 1.04 | 0.94 | 1.15 | 1.06 | 0.97 | 1.17 | |
| **PANAS-Positive affect** | 1.00 | 0.96 | 1.04 | 1.01 | 0.97 | 1.05 | |
| **PANAS-Negative affect** | 0.99 | 0.94 | 1.04 | 0.99 | 0.95 | 1.04 | |
| **HP5I-Antagonism** | 1.03 | 0.93 | 1.13 | 0.99 | 0.90 | 1.08 | |
| **HP5I-Impulsivity** | 1.14** | 1.04 | 1.25 | 1.12** | 1.03 | 1.22 | |
| **HP5I-Hedonic capacity** | 1.01 | 0.88 | 1.16 | 0.99 | 0.87 | 1.13 | |
| **HP5I-Negative affectivity** | 0.98 | 0.87 | 1.04 | 1.01 | 0.91 | 1.13 | |
| **HP5I-Alexithymia** | 1.00 | 0.91 | 1.10 | 1.01 | 0.92 | 1.11 | |
| **Age** | 0.99 | 0.98 | 1.00 | 0.99 | 0.98 | 1.00 | |
| **Gender ^d^** | 1.59* | 1.00 | 2.55 | 0.63* | 0.12 | 0.98 | |
| Statistical significance level: * *p* < 0.05 and ** *p* < 0.01  Logistic regression sensitivity analysis, with gambling problem severity as the outcome variable; levels of craving desire, anticipation, and relief, symptoms of anxiety and depression, propensity to experience positive and negative emotions, health-relevant personality traits, age, and gender simultaneously entered as predictors.  ^a^ Bivariat model [non-problem gamblers *n* = 850 vs. problem gamblers *n* = 203]: Naglekerke *R^2^* 0.49, Modelfit: χ²(14) = 387.756, *p* < 0.001  ^b^ Ordinal model [non-problem gambling *n* = 850 vs. at-risk gambling *n* = 119, problem gambling *n* = 84]: Naglekerke *R^2^* 0.47, Modelfit: χ²(14) = 438.715, *p* < 0.001  ^c^ OR = odds ratio; CI 95% for OR = 95% confidence interval for odds ratio  ^d^ Reference category female | | | | | | |  |
